# A Complicated Wound of the Leg

**Published:** 1894-02

**Authors:** John M. Eshleman

**Affiliations:** Parkesburg, Pa.


### A COMPLICATED WOUND OF THE LEG.

By John M. Eshleman, V.M.D.

On June 11th, 1892, I was called to attend a filly that had been caught in a wire fence. On arriving at the owner's stable I found a fine bay filly, 14 months old, well developed and in good condition.

The history, of course, was very brief, simply that the mare had gotten fast in the fence some time in the morning.

On examination I found the tissues of the near fore leg badly lacerated; the wound extended from above the carpal articulation down to the sesamoid bones; the skin was torn, lacerated and laid open the entire distance; the synovial bursa of the anterior and lateral extensor of the phalanges, the carpal articulation and the great sesamoid sheath, were all more or less complicated, so that the synovia was streaming down over the hoof; the opening in the bursa of the anterior extensor was large enough to admit two fingers; the metacarpal bone was scored by the barbs in many places; the suspensory ligament, the perforans and perforatus tendons were not exposed.

After my examination I advised the owner to have the filly destroyed, as it was my opinion she would not get well, but if she did she would be worthless; to this he and his family firmly objected, saying they would be satisfied with anything rather than have her killed. With their assurance of good care, I treated her in the following manner: Washed the parts with a bi-chloride solution, then made a large pad of oakum, saturated it in a 5 per cent. solution of creoline, and then applied it with light bandage to keep in place; this was done night and morning, during the day from 10 A. M. to 4 P. M. The wound was irrigated; the filly was kept in the slings for several weeks.

In about ten days all the synovial membranes had united except the lateral extensor, which did so about a week later, only to form an abscess which, after being opened, healed rapidly.

The greatest barrier to success now seemed to be "proud flesh," which was great, probably due to the low vitality of the tissues in this region.

When I last saw her, which was in the following October, her limb was about three times its natural size in the metacarpal region; she could raise her toe about six inches when traveling.

I do not write this with the idea that any of my fellow-practitioners may think I take any credit for the filly's recovery, but simply to show what may occur in such cases, and it might be worth trying in valuable brood animals.

PARKESBURG, PA.

